# Genetic Potential of the Biocontrol Agent *Pseudomonas brassicacearum* (Formerly *P. trivialis*) 3Re2-7 Unraveled by Genome Sequencing and Mining, Comparative Genomics and Transcriptomics

**DOI:** 10.3390/genes10080601

**Published:** 2019-08-09

**Authors:** Johanna Nelkner, Gonzalo Torres Tejerizo, Julia Hassa, Timo Wentong Lin, Julian Witte, Bart Verwaaijen, Anika Winkler, Boyke Bunk, Cathrin Spröer, Jörg Overmann, Rita Grosch, Alfred Pühler, Andreas Schlüter

**Affiliations:** 1 Center for Biotechnology (CeBiTec), Bielefeld University, Genome Research of Industrial Microorganisms, Universitätsstraße 27, 33615 Bielefeld, Germany; 2 Facultad de Ciencias Exactas, Departamento de Ciencias Biologicas, IBBM, Universidad Nacional de La Plata, Calle 115 y 47, 1900 La Plata, Argentina; 3 Leibniz-Institute DSMZ—German Collection of Microorganisms and Cell Cultures, Inhoffenstraße 7B, 38124 Braunschweig, Germany; 4 Leibniz-Institute of Vegetable and Ornamental Crops (IGZ), Plant-Microbe Systems, Theodor-Echtermeyer-Weg 1, 14979 Großbeeren, Germany

**Keywords:** *Pseudomonas brassicacearum*, genome mining, RNA sequencing, comparative genomics, biocontrol, plant-growth promotion, transcriptomics

## Abstract

The genus *Pseudomonas* comprises many known plant-associated microbes with plant growth promotion and disease suppression properties. Genome-based studies allow the prediction of the underlying mechanisms using genome mining tools and the analysis of the genes unique for a strain by implementing comparative genomics. Here, we provide the genome sequence of the strain *Pseudomonas brassicacearum* 3Re2-7, formerly known as *P. trivialis* and *P. reactans*, elucidate its revised taxonomic classification, experimentally verify the gene predictions by transcriptome sequencing, describe its genetic biocontrol potential and contextualize it to other known *Pseudomonas* biocontrol agents. The *P. brassicacearum* 3Re2-7 genome comprises a circular chromosome with a size of 6,738,544 bp and a GC-content of 60.83%. 6267 genes were annotated, of which 6113 were shown to be transcribed in rich medium and/or in the presence of *Rhizoctonia solani*. Genome mining identified genes related to biocontrol traits such as secondary metabolite and siderophore biosynthesis, plant growth promotion, inorganic phosphate solubilization, biosynthesis of lipo- and exopolysaccharides, exoproteases, volatiles and detoxification. Core genome analysis revealed, that the 3Re2-7 genome exhibits a high collinearity with the representative genome for the species, *P. brassicacearum* subsp. *brassicacearum* NFM421. Comparative genomics allowed the identification of 105 specific genes and revealed gene clusters that might encode specialized biocontrol mechanisms of strain 3Re2-7. Moreover, we captured the transcriptome of *P. brassicacearum* 3Re2-7, confirming the transcription of the predicted biocontrol-related genes. The gene clusters coding for 2,4-diacetylphloroglucinol (*phlABCDEFGH*) and hydrogen cyanide (*hcnABC*) were shown to be highly transcribed. Further genes predicted to encode putative alginate production enzymes, a pyrroloquinoline quinone precursor peptide PqqA and a matrixin family metalloprotease were also found to be highly transcribed. With this study, we provide a basis to further characterize the mechanisms for biocontrol in *Pseudomonas* species, towards a sustainable and safe application of *P. brassicacearum* biocontrol agents.

## 1. Introduction

Productivity in agriculture has been increased, for example, by application of fertilizers and pesticides. However, there is a serious concern that land use intensification in agriculture results in large-scale ecosystem degradation, loss of crop productivity and reduction of biodiversity [1]. Plants are associated with a high number of microbes of which some are pathogenic while others feature plant beneficial properties. Plant diseases like bare patch of cereals, brown patch of turf, root canker of lucerne and black scurf of potato caused by plant pathogens as for example the fungus *Rhizoctonia solani* can lead to yield losses. It was estimated that such soilborne pathogens are responsible for up to 25% yield losses in relevant crops worldwide [2]. Effective strategies to control soilborne pathogens are limited today. In terms of disease control, it is well-documented that beneficial plant-associated microbes can protect plants from disease infestations and prevent the deficiencies caused by exclusive reliance on pesticides [3,4]. Biocontrol, the concept of using beneficial microbes for pathogen control as strategy for sustainable agriculture is established and has been studied intensively over the last years as an alternative for the use of chemical pesticides. Microbial biocontrol agents (BCAs) have been shown to be involved in direct suppression of pathogens [5], as well as in inducing systemic resistance in plants to enhance tolerance to different environmental stresses [6]. These biocontrol characteristics have been linked to their ability to produce compounds featuring antimicrobial activity, siderophores and chelators, exoproteases, phosphorus solubilization, as well as plant growth regulation, stimulation and signaling molecules [7,8].

In a study addressing the effect of long-term farming practices, the genus *Pseudomonas* has been shown to be abundant in agricultural soils [9]. Many soilborne pseudomonads have the potential to suppress plant pathogens and promote plant growth and health, making them candidates as effective BCAs [3]. Especially the group of fluorescent *Pseudomonas* species is studied intensively and used as effective biocontrol agent against various plant pathogens [10]. Members of this group have great potential in biocontrol because of their ability to produce secondary metabolites relevant in suppression of pathogens [11,12,13,14,15].

The species *Pseudomonas brassicacearum* also comprises plant-associated strains obtained from the root surface (rhizoplane), the zone around the root (rhizosphere) as well as endophytes of different plant hosts [16,17,18,19,20,21]. Most known *P. brassicacearum* strains were described as potentially phyto-beneficial; further they have been shown to produce antimicrobial compounds and feature activity against phytopathogenic microbes [16,17,18,19,20,21]. The type strain of the species is *P. brassicacearum* DBK11 (CFBP 11706${}^{T}$/DSM 13227${}^{T}$) [16] but only its 16S rDNA sequence is available so far. Therefore the genome of *P. brassicacearum* subsp. *brassicacearum* NFM421 [18] was chosen as reference for genome-based studies regarding this species [19,22].

Gene clusters such as the *phlABCDEFGH* and *hcnABC* operons responsible for the biosynthesis of 2,4-diacetylphloroglucinol (DAPG) and hydrogen cyanide (HCN) were detected in the genomes of *P. brassicacearum* strains. DAPG and HCN have been linked to biocontrol traits [23]. Furthermore, genes involved in the production of 1-aminocyclopropane-1-carboxylate deaminase (*acdS*) and pyrroloquinoline-quinone (*pqqABCDEF*) were predicted in genomes of *P. brassicacearum* strains and have been associated with plant growth-promotion (PGP). Also, different secretion systems, genes for the biosynthesis of different volatile compounds and exoproteases have been identified and are likely to contribute to the biocontrol potential of this species.

Subject of this study is the strain *Pseudomonas brassicacearum* 3Re2-7, formerly known as *P. trivialis* 3Re2-7 [24] and *P. reactans* 3Re2-7 [25]. It was originally isolated from the endorhiza of potato in a study addressing the screening of potential biocontrol agents in large scale [25]. The strain 3Re2-7 was selected as successful biocontrol strain able to suppress soilborne pathogens such as *Rhizoctonia solani* on lettuce and sugar beet [26]. Moreover, it was shown to be able to produce proteases and siderophores and to harbour the *phlD* gene which is involved in the production of DAPG. In a follow-up study involving 20 isolates which are antagonistic to *R. solani*, it was shown to be one of the two most effective strains and also featured a positive effect on plant growth in vitro. At that time, it was classified as *Pseudomonas reactans*, based on its 16S rDNA sequence and fatty acid methyl ester profile [25,26]. Further, being unlikely to cause diseases in humans, it was placed into the low risk group 1 (classification according to Technical Rules for Biological Agents (TRBA) from the German Federal Institute for Occupational Safety and Health, TRBA 466 “Classification of prokaryotes (bacteria and archaea) into risk groups”). In 2006, it was reclassified as *P. trivialis*. Moreover, it was shown to produce 12 different small volatile organic compounds (VOCs), most representing unidentified compounds but undecadiene, undecene and (benzyloxy)benzonitrile were identified [24]. Also, an antagonistic effect of these VOCs on *R. solani* has been shown in this study. In another study, a low and short term effect of strain 3Re2-7 on the indigenous plant-associated microbial community has been proved [27]. The strain 3Re2-7 is commercially available as growth enhancing crop inoculant.

Like most genome sequences nowadays, those of *P. brassicacearum* strains, have been published only in short formats (Short Genome Communications and Genome Announcements), lacking both a comprehensive genomic and a comparative study of the species [18,19,20,21,22].

In this study, we established the complete genome sequence of the BCA *P. brassicacearum* 3Re2-7. We aim to give an overview and classification of the genetic biocontrol determinants encoded in this biocontrol agent by mining its genome sequence. Using various bioinformatics tools and databases, we screen for known genes and features but also identify potentially new features by similarity, motif structures and cluster structures. By implementing comparative genomics of publicly available complete and draft genome sequences of *P. brassicacearum* species, we provide an overview of the *P. brassicacearum* taxon. We thereby focus on strain 3Re2-7 to elucidate its unique genetic potential. Further, we apply RNA-Seq to monitor the transcription of biocontrol genes. By combining bioinformatics approaches of genome mining, comparative genomics and transcriptomics, we aim to improve the understanding of biocontrol traits in *P. brassicacearum* species. The analyses provide the basis to further characterize the mechanisms for niche adaptation, antagonism towards phytopathogenic fungi, as well as plant growth promotion in *P. brassicacearum*.

## 2. Results and Discussion

### 2.1. Genome Sequencing and General Genome Features of *P. brassicacearum* 3Re2-7

**Figure 1:**
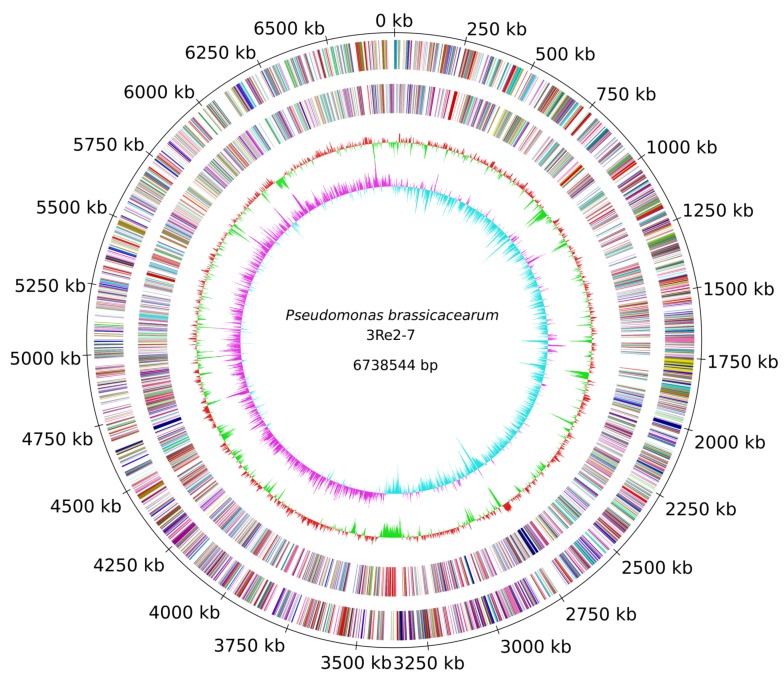
Circular representation of the *P. brassicacearum* 3Re2-7 chromosome. From the outer to the inner concentric circle: Circle 1, genomic position in kb (total 6,738,544 bp); the replication initiation gene *dnaA* was selected as first gene of the circular chromosome. Circles 2 and 3, predicted protein-coding sequences (CDS) on the forward [transcribed clockwise] (outer part) and the reverse [transcribed counter clockwise] (inner part) strand colored according to the assigned COG classes. Circle 4, GC content showing deviations from the average. Circle 5, GC skew.

**Figure 2:**
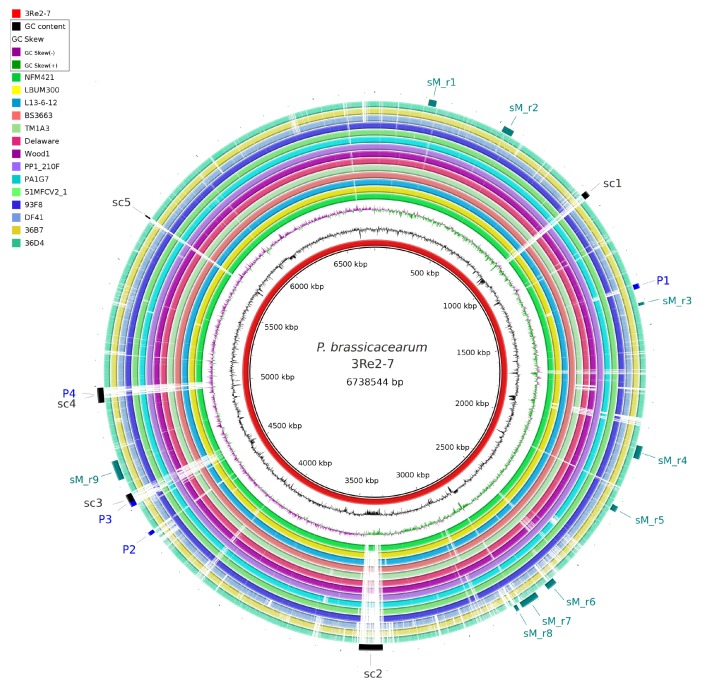
BLAST comparison of *P. brassicacearum* genomes. Circular representation of the similarity between *P. brassicacearum* genomes listed in Table 1 with strain 3Re2-7 as reference (black innermost ring). The innermost graphs depict GC content (black) and GC skew (purple/green) followed by concentric rings of query sequences colored according to BLAST identity; gaps in the rings represent regions of low or no similarity. On the outermost ring, clusters of 3Re2-7 specific genes are indicated in black, abbreviated with sc* (Singleton cluster 1-5), gene regions related to biosynthesis of secondary metabolites detected with antiSMASH are indicated in teal and abbreviated with sM_r* (secondaryMetabolism_region 1-9) and detected phage sequences in blue (P1-P4). BLAST analysis and generation of this comparative view was done using BRIG [31].

In order to establish the genome sequence of the biocontrol strain *P. brassicacearum* 3Re2-7 and enable genome-based analyses, its genome was sequenced using a combination of PacBio and Illumina technologies. The obtained reads were assembled into one high-quality continuous contig of 6,738,544 bases, featuring a GC content of 60.8% and an average coverage of 104-fold. Figure 1 shows a circular representation of the 3Re2-7 chromosome. In total, 6267 genes, of which 5992 are annotated as protein-encoding sequences (CDSs), 190 pseudo genes, 65 tRNAs, 4 ncRNAs and 16 rRNA genes could be predicted. Four prophage regions were identified using PHASTER [28], of which three regions were predicted to be complete (P1, *Synechococcus* phage S-CAM7, NCBI accession NC_031927; P3, *Pseudomonas* phage YMC11/02/R656, NC_028657 and P4, *Enterobacter* phage Arya, NC_031048) and one region (P2, *Pseudomonas* phage phiPSA1, NC_024365) is incomplete. The localizations of these prophage regions are indicated in Figure 2.

No antimicrobial resistance genes could be detected using ResFinder-3.1 [29]. Moreover, neither virulence genes, resistance genes, pathogen-associated genes nor homologs of these were detected by means of IslandViewer 4 [30].

### 2.2. Taxonomic Classification Based on Whole-Genome and Core Gene Analyses

In 2002, the strain 3Re2-7 was first classified as *Pseudomonas reactans* by fatty acid methyl ester analysis and 16S rDNA sequencing, and then in 2004 reclassified according to its 16S rDNA sequence as *P. trivialis* [25,26,32]. The intrageneric similarity of 16S rRNA gene sequences between species of the genus *Pseudomonas* is very high (>98.5%, [33]). Therefore, the resolution of 16S rRNA gene sequence similarity values is not high enough for *Pseudomonas* species delineation [11,34]. Based on the whole genome sequence, we revised the taxonomic classification of the strain 3Re2-7 to *P. brassicacearum* using the high-resolution classification approaches of Average Amino-acid Identity (AAI) and in silico DNA-DNA hybridization (DDH). These analyses showed, that AAI values are at 85.00% and 99.59% for identities with *P. trivialis* DSM 14937${}^{T}$ and *P. brassicacearum* subsp. *brassicacearum* NFM421, respectively. Accordingly, the in silico DDH estimate is at 25.30% with respect to *P. trivialis* DSM 14937${}^{T}$ and 96.10% to *P. brassicacearum* subsp. *brassicacearum* NFM421. Therefore, the strain 3Re2-7 can be classified as *P. brassicacearum* rather than *P. trivialis*.

**Figure 3:**
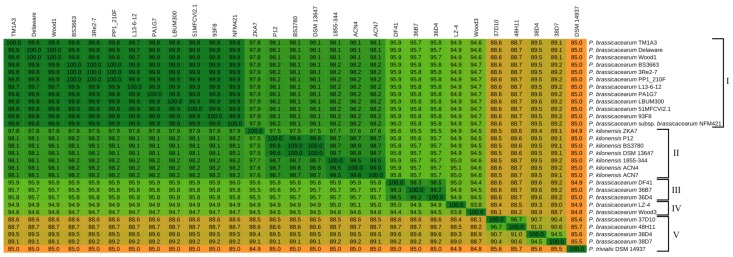
Average Amino-acid Identity (AAI) matrix of genome sequenced *P. brassicacearum* and *P. kilonensis* strains. Emerging clusters/groups are indicated with brackets and numbered I-V. The protein sequences of orthologous core genes of the genomes were analyzed for their mean percent identity values. The values were calculated within the EDGAR2.0 platform [42].

Further, pairwise Average Amino-acid Identities (AAIs) of all publicly available *P. brassicacearum* strains (as listed in Table 1) were calculated. Additionally, all publicly available *P. kilonensis* strains were included in the AAI analysis, since *P. kilonensis* has previously been suggested as the ’junior synonym’ to *P. brassicacearum* [35]. As shown in Figure 3, calculated pairwise AAIs show values of 99.8% and higher for twelve very closely related strains including the strain 3Re2-7 and the representative *P. brassicacearum* strain NFM421 (cluster I including TM1A3, Delaware, Wood1, BS3663, 3Re2-7, PP1_210F, L13-6-12, PA1G7, LBUM300, 51MFCVI2.1, 93F8 and NFM421).

The *P. kilonensis* strains form a distinct cluster (Figure 3, cluster II) but feature high AAI values of 97.5% and higher to cluster I.

A third separate cluster is formed by the strains DF41, 36B7 and 36D4 (Figure 3, cluster III). These three strains are more distinct from the highly similar *P. brassicacearum* and *P. kilonensis* clusters but still above the proposed 95% species threshold [36,37]. Even though AAI values of strains LZ_4 and Wood3 to the representative *P. brassicacearum* strain NFM421 are slightly below the species threshold, they are still relatively high (94.9% and 94.7%, respectively, Figure 3, cluster IV). They might still be considered as *P. brassicacearum* species. In contrast, AAI values of the strains 37D10, 48H11, 38D7 and 38D4 to the *P. brassicacearum* representative strain are clearly below the 95% species threshold (Figure 3, group V) and therefore their species allocation should be rethought. Thus, these four strains were not included in the detailed comparative analysis.

**Figure 4:**
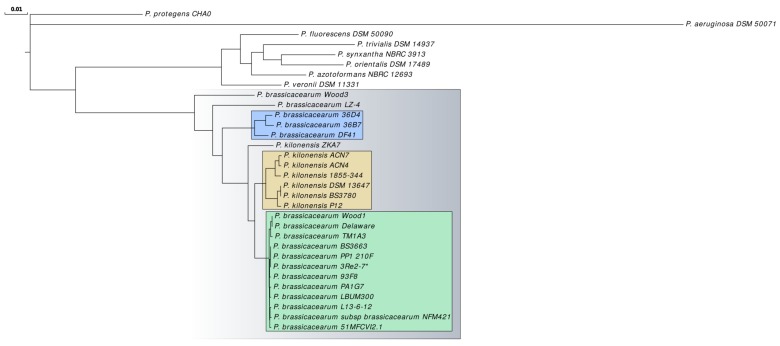
The phylogenetic relationship of *P. brassicacearum* species relative to other biocontrol *Pseudomonas* species. As outgroup, the type strain of the genus, *P. aeruginosa* DSM 50071${}^{T}$, was included. The tree is based on 2331 core gene products. Selected clusters are indicated by colored boxes. Strain *P. brassicacearum* 3Re2-7 studied here is marked with an asterisk. The phylogenetic analysis was performed within the EDGAR 2.0 platform [42]. The bar indicates 1 substitutions per 100 positions.

The constructed phylogenetic tree (Figure 4) based on 2331 core genes shows the position of the analyzed *P. brassicacearum* strains relative to type strains of other biocontrol *Pseudomonas* species (*P. fluorescens* DSM 50090${}^{T}$, *P. veronii* DSM 11331${}^{T}$, *P. azotoformans* NBRC 12693${}^{T}$, *P. trivialis* DSM 14937${}^{T}$, *P. orientalis* DSM 17489${}^{T}$ and *P. synxantha* NBRC 3913${}^{T}$). A distinct cluster is formed by the *P. brassicacearum* species, which as expected includes the *P. kilonensis* strains. The subclusters that emerged in the AAI analysis also appear in the phylogenetic tree (Figure 4).

Interestingly, *P. brassicacearum* strains with very similar GC contents clustered together independently of the method used (Table 1, Figure 3 and Figure 4). This observation suggests an evolutionary association of GC content and phylogenetic distance within the species. The exceptional high similarity between *P. brassicacearum* strains may reflect adaptations to similar environmental conditions. Unfortunately, metadata reporting is inconsistent. For some strains the exact origin is reported unspecifically or unknown. Consistent and specific metadata would allow us to draw conclusions or formulate hypotheses regarding niche adaptation.

### 2.3. Comparative Genomics Revealed Genes Unique to *P. brassicacearum* 3Re2-7

To assess the unique genetic potential of strain 3Re2-7, a comparative analysis including publicly available genomes of in total 16 *P. brassicacearum* strains confirmed by AAI analyses was performed using the software platform EDGAR [42]. The core genome of the *P. brassicacearum* species consists of 3556 CDSs with *P. brassicacearum* subsp. *brassicacearum* NFM421 used as reference. For *P. brassicacearum* 3Re2-7, 105 unique genes without any orthologous genes in the compared genomes were detected. Of these 105 singletons, 78 were annotated as genes encoding hypothetical proteins and 27 have predicted functions. Some of these singletons are clustered within the genome (see their regions indicated in Figure 2). During the detailed analysis, five regions emerged, in which clustered singletons occur. A striking deviation in the GC content to the remaining genome was observed in the genomic regions where these clusters are localized (Figure 2). These regions may represent functional clusters of so far unknown function. The smallest cluster comprises four singleton genes and the largest 25. Cluster_3 and cluster_4 correspond to the location of the detected prophage regions P3 and P4, respectively.

Cluster_1 comprises 13 singleton genes that encode hypothetical proteins with unknown function and four singleton genes with the functions listed in Table 2. Metallohydrolases are enzymes that catalyze the hydrolysis of ester and amide bonds [43], while nucleotidyltransferases transfer nucleotides from one compound to another [44]. An adenylating protein similar to ThiF has been shown to be involved in the biosynthesis of the unusual siderophore thioquinolobactin in *Pseuodomonas fluorescens* ATCC17400 [45]. The DUF4935 domain shows homology to other PIN-like families and is found in the N-terminal region of uncharacterized proteins. It contains several conserved acidic residues critical for chelating metal ions [46]. In the genetic context, also a non-singleton ferric iron uptake transcriptional regulator gene (ELZ14_04245) is present. The regulator was shown to repress and also activate siderophore synthesis in pathogens [47]. According to IslandViewer 4 [30], the region enclosing cluster_1 is a genomic island (supported by three of four prediction methods). In between the singleton genes, a non-singleton gene encoding an integrase (ELZ14_04135) is located. Integrases are known to mediate integration of integrative conjugative elements (ICEs; a.k.a. conjugative transposons) [48]. ICEs are modular mobile genetic elements (MGEs) integrated into a host genome and have a broad range of associated phenotypes, for example, antibiotic resistance, symbiosis, pathogenesis, bacteriocin synthesis and biofilm formation [48]. Further, there is evidence for a toxin-antitoxin system encoded in cluster_1 (ELZ14_04230, ELZ14_03880-03895). A toxin-antitoxin system is an accessory function typical of MGEs that most probably promotes the maintenance of an ICE [49]. Due to these findings, it could be possible that this putative MGE encodes enzymes for the synthesis of an unknown siderophore.

The largest cluster (cluster_2) includes 14 hypothetical proteins and 11 annotated genes with the functions listed in Table 3. Helicase IV catalyses ATP dependent unwinding of double stranded DNA to single stranded DNA [50], while endonucleases are enzymes that cleave the phosphodiester bond within a polynucleotide chain. A relation of some proteins containing the CHAT (Caspase HetF Associated with Tprs) domain to peptidases in peptidase clan CD that includes the caspases is known [51,52]. The DUF3742 domain family is found in bacteria and is of so far unknown function [51]. The haloacid dehalogenase-like hydrolase (HAD) superfamily is a large group of proteins with diverse substrate specificities whose members are involved in the cleavage of carbon-halogen bonds, phosphatase, phosphonatase and phosphoglucomutase reactions [53]. RNA-directed DNA polymerase (reverse transcriptase) occurs in a variety of mobile elements, including prokaryotic retroelements and bacterial msDNAs [51]. The enzyme synthesizes DNA on a RNA template for integration into the host genome and exploitation of a host cell [51]. Interestingly, this cluster was reported as genomic island supported by four prediction methods integrated in IslandViewer 4 [30]. Some genes included in this region (not reported as singletons, ELZ14_15120, ELZ14_15125, ELZ14_15150-75, ELZ14_15190-205 and ELZ14_15250) have a function associated with integrative conjugative elements (ICEs). Also a relaxase (ELZ14_15105) is encoded in cluster_2. Relaxases perform the initial step in the ICE transfer mechanism [48]. Type I and type VI secretion as well as type IV conjugative transfer system components are also present in the region of singleton cluster_2 (ELZ14_14955, ELZ14_15020, ELZ14_15025 and ELZ14_15195) and suggest the functionality of the putative ICE [48]. Taken together these results with the predicted protein functions, cluster_2 might be of horizontal origin and have a possible function in the biocontrol context and contribute to bacterial fitness and competitiveness in the endorhiza.

The small cluster_5 shown in Table 4 was predicted to encode two hypothetical proteins (ELZ14_26355 and 26360), a DNA helicase (ELZ14_26365) and a GNAT family N-acetyltransferase (ELZ14_26370). DNA-helicase unwinds the two strands in a DNA double helix. GCN5-related N-acetyltransferases family (GNAT) N-acetyltransferases transfer an acetyl group from acetyl-CoA to a large array of substrates, from small molecules such as aminoglycoside antibiotics to macromolecules [54]. These singletons also are located in a region predicted as genomic island by IslandViewer 4 [30] (supported by three prediction methods). A functional prediction for this region can not be deduced.

*P. brassicacearum* 3Re2-7 was isolated from the endorhiza of potato and therefore most probably has evolved mechanisms to sustain in competitive environments like soil, rhizosphere and endorhiza. By combining genome mining with comparative genomics, we were able to find gene clusters specific to strain 3Re2-7 with the potential to be related to niche adaptation or biocontrol. Our results show, that even though the genomes of *P. brassicacearum* strains are very similar, they still have unique traits that are interesting targets for the detection of new microbial gene clusters of biotechnological potential, that need experimental confirmation.

### 2.4. Genome Mining of *P. brassicacearum* 3Re2-7 Revealed Biocontrol Determinants

To examine putative genetic determinants involved in biocontrol characteristics of *P. brassicacearum* 3Re2-7, different genome-mining tools were applied. Putative biocontrol genes involved in the biocontrol activity of this strain were identified in the genome and are discussed in the following sections. Genes predicted to encode functions in secondary metabolite and antibiotic biosynthesis (Table 5), induced systemic resistance and plant growth promotion (Table 6), pathogen inhibition (Table 7) and other traits of importance in a competitive environment (Table 8) were identified. The localization of selected genetic features in the 3Re2-7 genome is indicated in Figure 2.

#### 2.4.1. Secondary Metabolism and Antibiotics

Using antiSMASH, nine regions predicted to encode secondary metabolite synthesis genes were detected in the 3Re2-7 genome. These include three antiSMASH type ’NRPS’ regions (nonribosomal peptide synthetase, region sM_r4, sM_r7 and sM_r9) which are described in Subsection ’2.4.3. Pathogen inhibition’.

Region sM_r1 of the type ’NRPSfragment’ (cluster with similarity to a NRPS fragment) showed a high similarity to the so-called Mangotoxin biosynthetic gene region (*mgo*-operon) that has been found in both pathogenic as well as beneficial *Pseudomonas* species [55,56]. Most probably its product acts as a regulator of different functions or as a *Pseudomonas*-specific switch for secondary metabolite production, rather than a toxin, but further details remain to be explored [55,56]. Moreover, no pathogenicity factor or compound has been linked to *P. brassicacearum* 3Re2-7 and the absence of any virulence, resistance and pathogen-associated genes was confirmed by means of Genomic IslandViewer 4 [30]. The predicted gene region sM_r1 comprises 28 genes. The core biosynthetic gene most probably is ELZ14_01120, encoding an amino acid adenylation domain-containing protein. ELZ14_01145 was predicted to be an additional biosynthetic gene. Its gene product carries an EAL domain which is named after its conserved residues and is found in diverse bacterial signalling proteins [51]. Further, the region involves several transport-related and regulatory genes.

A significant similarity to the aryl polyene biosynthetic gene cluster from the γ-proteobacterium *Vibrio fischeri* (BGC0000837, 40% of genes show similarity) was reported for region sM_r2. An important role in Gram-negative cell biology is thought to be played by the products of gene clusters from the large and unexplored aryl polyene biosynthetic gene cluster family [57].

While antiSMASH predicted one region of type ’Bacteriocin’ (region sM_r3), a screening with BAGEL4 yielded four putative bacteriocin gene clusters featuring similarity to genes coding for the synthesis of a microcin and three class III bacteriocins (colicin). Region sM_r3 does not show significant similarities to known clusters but one of the predicted core biosynthetic genes of the bacteriocin region sM_r3 detected by antiSMASH encodes a DUF629 domain-containing protein (ELZ14_06450). This domain of yet unknown function has been observed in several plant proteins. Therefore, this region is an interesting target for mutant analyses in combination with plant interaction studies.

By similarity to the beta-lactone fengycin biosynthesis cluster, region sM_r5 was predicted to represent the type ’betalactone’. Fengycin is known to be involved in biocontrol activity of *Bacillus* strains [58].

Further, the type III polyketide synthase (PKS) region (region sM_r6) predicted by antiSMASH is identical to the 2,4-diacetylphloroglucinol (2,4-DAPG) biosynthetic gene cluster from *Pseudomonas fluorescens*. Thereby, all genes required for the synthesis of the antibiotic 2,4-DAPG are present (Table 5).

The region assigned to the type ’Butyrolactone’ (region sM_r8) showed no significant hits to known secondary metabolite clusters. By means of further detailed analysis, the gene operon needed for the production of the secondary metabolite hydrogen cyanide (*hcnABC*) was annotated.

A further determinant for production of secondary metabolites is acetohydroxyacid synthase (AHAS, EC 2.2.1.6). AHAS is the key enzyme in branched chain amino acid biosynthesis in bacteria. In the 3Re2-7 genome, the genes *ilvH* (ELZ14_26510) and *ilvI* (ELZ14_26515) coding for the small and large subunit of AHAS, respectively, were predicted. Also, the gene *ivlC* (ELZ14_26505) was annotated, coding for ketol-acid reductoisomerase (KARI, EC 1.1.1.86). Since AHAS and KARI are able to form precursors for a number of secondary metabolites such as cyanogenic glycosides, glucosinolates and acyl-sugars, both enzymes belong to the KEGG pathways ’Biosynthesis of secondary metabolites’ (ec01110) and ’Biosynthesis of antibiotics’ (ec01130) [50].

#### 2.4.2. Induced Systemic Resistance and Plant Growth Promotion

*P. brassicacearum* 3Re2-7 has the metabolic potential to produce proteins involved in the synthesis of volatiles. Amongst others, bacterial volatiles such as 2,3-butanediol have been linked to Induced Systemic Resistance (ISR). In the rhizosphere, volatiles elicit root exudates which selectively affect different bacterial species [59,60]. Also, 2,3-butanediol has been shown to play a role as a bacterial protectant. It protects bacterial cells against putative harmful plant root exudates and low pH [61]. The translated protein sequence of the gene ELZ14_17085 shows 100% identity to the *Pseudomonas* multispecies protein sequence of butanediol dehydrogenase. This enzyme is involved in the production of the volatile 2,3-butanediol [61].

Genetic determinants possibly involved in plant growth promotion include the *acdS* gene coding for 1-aminocyclopropane-1-carboxylate (ACC) deaminase (ELZ14_11285). ACC is the immediate precursor of the phytohormone ethylene. Through deamination, ACC gets broken down to ammonia and α-ketobutyrate, which can be further metabolized by bacteria. At the same time, ACC deaminase lowers plant ethylene levels. This mechanism has been observed in plant growth promoting bacteria to facilitate plant growth. Also, the *acdS* gene product has been shown to have a positive impact on plant growth under different environmental stresses [62]. Pairwise amino acid sequence comparison of the predicted protein sequence of the *acdS* gene (ELZ14_11285) in the 3Re2-7 genome revealed a complete match to the aminocyclopropane-1-carboxylate deaminase/D-cysteine desulfhydrase family protein sequence of different *Pseudomonas* species, suggesting that the putative *acdS* gene in the 3Re2-7 genome is functional.

*P. brassicacearum* 3Re2-7 further encodes riboflavin synthase (EC 2.5.1.9, ELZ14_27880), which catalyzes the final reaction of riboflavin biosynthesis. Riboflavin stimulates plant growth and is known to function as protectant/elicitor of plant defense [63,64].

A region encoding a Type III secretion system (T3SS) was predicted (ELZ14_29730-29880). T3SSs are found in Gram-negative bacterial pathogens and symbionts and enable bacteria to inject effector proteins into the host cell [65]. Further, T3SSs are believed to have a significant role in the biology of plant growth promoting rhizobacteria such as *P. fluorescens* SBW25 [66].

Many plant-beneficial soil bacteria are capable of solubilizing phosphorus by synthesizing organic acids and acid phosphatases, making it available to plants [67]. Screening for genes related to phosphate solubilization uncovered a phytase gene (ELZ14_15680) and several unspecific phosphatase genes (listed in Table 6). Moreover, strain 3Re2-7 is most probably able to solubilize inorganic phosphate, since in its genome the *gcd* gene coding for a glucose/quinate/shikimate family membrane-bound pyrroloquinoline quinone (PQQ)-dependent dehydrogenase was predicted (ELZ14_24970). This enzyme together with its cofactor PQQ catalyzes the production of gluconic acid [68]. All six genes of the operon for the pyrroloquinoline quinone coenzyme biosynthesis were observed, including the *pqqC* gene coding for the PQQ synthase (*pqqABCDEF*, ELZ14_28615-28640). The presence of a phytase and several phosphatases suggests that *P. brassicacearum* 3Re2-7 is able to solubilize high amounts of phosphorus, which might add to its plant beneficial potential.

#### 2.4.3. Pathogen Inhibition

The biosynthetic pathway for the production of aromatic amino acids majorly contributes to the broad functional spectrum of organisms in nature. Various pigments, siderophores, signaling compounds, defense metabolites, structural compounds, antibiotics and other secondary metabolites are derived from this pathway [69]. In *Pseudomonas* species, this pathway starts with chorismate [69]. Chorismate can be transformed into anthranilate, by the action of anthranilate synthase (TrpE/TrpG), which is encoded in the genome (ELZ14_28410/ELZ14_28400). Anthranilate synthase is an enzyme of the menaquinone, siderophore and tryptophan (MST) class [70]. Siderophores can enhance iron acquisition and suppress target pathogens in the rhizosphere through iron competition and are therefore of importance for biocontrol. The *pchDCBA* operon is required for the biosynthesis of the high-affinity siderophore salicylate, which is also an intermediate in the biosynthetic pathway of pyochelin, another siderophore [71]. Of the *pchDCBA* operon, the gene coding for isochorismate lyase PchB (ELZ14_13330 *pchB*) was predicted in the genome of *P. brassicacearum* 3Re2-7. Also, the neigboring gene is annotated as isochorismatase family protein (ELZ14_13335), which is involved in the biosynthesis of several siderophores, such as 2,3-dihydroxybenzoylglycine, enterobactin, bacillibactin and vibriobactin [72].

For the antiSMASH Nonribosomal Polyketide Synthase (NRPS)-type regions sM_r4, sM_r7 and sM_r9 (Figure 2), significant similarities to known pyoverdine, serobactins, cupriachelin, taiwachelin, bleomycin, viscosin and delftibactin biosynthetic gene regions were reported. Region sM_r9 encoding an unknown non-ribosomal polyketide synthetase, harbors the *pvdA* gene (ELZ14_21210). Its gene product ornithine 5-monooxygenase catalyzes the first step in the biosynthesis of all hydroxamate-containing siderophores, such as pyoverdine. It further includes the putative core biosynthetic genes predicted to code for three non-ribosomal peptide synthases (ELZ14_21260, ELZ14_21285 and ELZ14_21295), an amino acid adenylation domain-containing protein (ELZ14_21290) and a peptide synthase (ELZ14_21300). Also, transport related genes (ELZ14_21220-21230, ELZ14_21255 and ELZ14_21280) were annotated in this region, suggesting an export mechanism. Further, genes of the cluster show similarity to genes of a pyoverdine NRPS gene cluster from *P. protegens* (BGC0000413). These results suggest that this region encodes an unknown NRPS and that its product might be a siderophore. Detailed analyses of the modular architectures of PKS/NRPS clusters often enable the prediction of the produced compounds, even if the biological function or bioactivity remains unknown [8]. The predicted core structure of the compound produced by the NRPS is shown in Supplementary File S1. Its predicted structure resembles the antibiotic peptide phosphinothricin tripeptide (NOR00670) listed in the Norine database [73].

Region sM_r4 of the regions identified by antiSMASH also includes a core biosynthetic gene whose gene product was annotated as non-ribosomal peptide synthetase (ELZ14_09165). The most similar known cluster is also the *P. protegens* pyoverdine biosynthetic gene cluster BGC0000413.

The third predicted NRPS type cluster located in region sM_r7, has five annotated core biosynthetic genes, predicted to encode four non-ribosomal peptide synthetases (ELZ14_12310-12325) and a peptide synthase (ELZ14_12335). Transport-related genes are also present in this region (ELZ14_12285, ELZ14_12305 and ELZ14_12345). The cluster is most similar to the known cupriachelin cluster from *Ralstonia eutropha* (BGC0000330) and the predicted product has similarity to the peptide chrysobactin (NOR00210), which shows siderophore activity.

On that basis, it can be hypothesized that *P. brassicacearum* 3Re2-7 is able to produce siderophores.

#### 2.4.4. Other Biocontrol Features

Exopolysaccharides are known to aid colonization and biofilm persistence, which is especially important in harsh and competitive environments like soil and rhizosphere [74]. The *P. brassicacearum* 3Re2-7 chromosome contains a cluster comprising twelve genes (ELZ14_25290-350) predicted to encode functions related to the biosynthesis of the exopolysaccharide alginate. Further, it may have the genetic potential to produce the exopolysaccharide levan, since it was predicted to encode a glycosyl hydrolase 68 (GH68) family protein (ELZ14_27635). The GH68 family includes several bacterial levansucrase enzymes.

*P. brassicacearum* 3Re2-7 encodes genes for the biosynthesis of various bacterial cell envelope components, that is, lipopolysaccharides (LPS) (ELZ14_02690, ELZ14_02695, ELZ14_02715, ELZ14_04750, ELZ14_04755, ELZ14_04760, ELZ14_20190). LPS can serve as a barrier to many antibiotics and changes in lipid composition may enable microorganisms to maintain outer membrane functions while facing environmental fluctuations. Interestingly, *P. brassicacearum* 3Re2-7 encodes a LTA synthase family protein predicted to be involved in the biosynthesis of lipoteichoic acid (ELZ14_31170). This is atypical for Gram-negative bacteria. In Gram-positive bacteria, teichoic acid is believed to i.a. function in biofilm formation and host tissue adhesion [75]. Most probably, the production of lipoteichoic acid adds to the rhizosphere competence of strain 3Re2-7.

Gene ELZ14_17635 is a homolog of *acnB*, coding for aconitase B. The production of aconitase B has been shown to be required for optimal growth sustainment of pathogenic *Xanthomonas campestris* in pepper plants. Since *P. brassicacearum* 3Re2-7 is a plant growth promoting bacterium, this trait most probably indicates a mechanism to enhance competitiveness in the rhizosphere or endorhiza. Also, two *mqo*-homologous genes (ELZ14_13780, ELZ14_26110) coding for malate:quinone oxidoreductase were identified. This enzyme has been hypothesized to be involved in growth adaptation to the host environment in pathogenic *P. syringae* [76]. In the beneficial *P. brassicacearum*, it might be advantageous for the endophytic lifestyle. Further putative biocontrol determinants are listed in Table 8.

*P. brassicacearum* 3Re2-7 encodes the potential to synthesize a great variety of biocontrol related compounds, including secondary metabolites, bacteriocins and siderophores. Summarized, corresponding genetic determinants detected by genome mining make *P. brassicacearum* 3Re2-7 a promising biocontrol agent.

### 2.5. Transcriptome Sequencing of *P. brassicacearum* 3Re2-7 Confirms Expression of Biocontrol Related Genes

**Figure 5:**
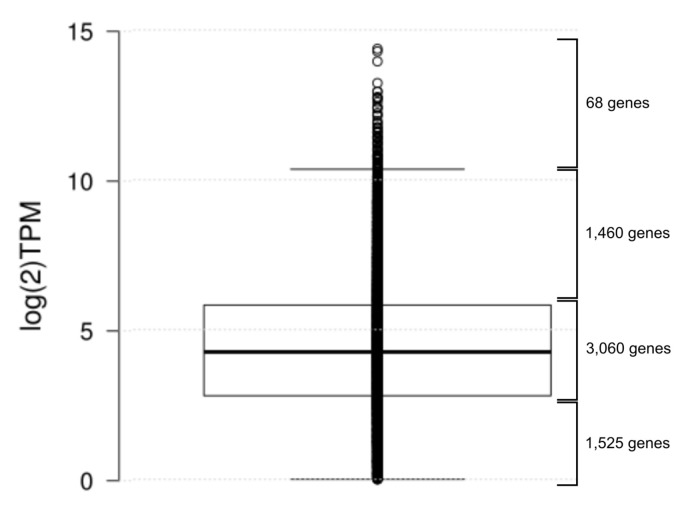
Box-Whisker plot of the log2-transformed Transcript per Million (TPM) values of transcribed genes. The center line shows the median (4.25), box limits indicate the 25th and 75th percentiles as determined by Tukey (2.79 and 5.82, respectively). Whiskers extend up to 1.5 times the interquartile range from the 25th and 75th percentiles (0.00 and 10.35, respectively), data points are plotted as open circles, n = 6113 sample points.

In order to verify transcription of predicted biocontrol-related genes and to detect genes organized in operons, whole-transcriptome sequencing was applied. Transcriptomic analyses in the context of biocontrol properties often implement RNA-Seq to reveal responses of a BCA to a pathogen [15,77,78]. Phenotypic variation of *Pseudomonas* species has been elucidated as relevant in competitive root colonization and biological control of phytopathogens [79,80]. The production of exo-enzymes and secondary metabolites in phase variation has been shown to affect colonization behaviour and biocontrol activity of rhizosphere bacteria [80]. Therefore, under laboratory conditions, some genes may be silent due to the absence of appropriate triggers. Since these could be activated by, for example, co-cultivation, we included transcriptomic data from an co-cultivation of *P. brassicacearum* 3Re2-7 with the pythopathogenic fungus *Rhizoctonia solani* AG1-IB. Transcriptome sequencing of *P. brassicacearum* yielded in total 46,076,764 reads amounting to 3,428,193,102 bases. With BBMap, 40,328,306 assignable mappings were generated. Feature coverage analysis revealed, that 4983 of the 6267 predicted genes are covered considering a minimum covered percentage of 90% and a minimum count of ten reads per gene. 6113 genes were considered as transcribed, since their Transcript Per Million (TPM) count was above the determined cutoff (TPM > 1). No genes were reported as differentially expressed (p-adjusted value < 0.05 and log2 fold change > 2). A possible explanation might be, that the biocontrol features making *P. brassicacearum* 3Re2-7 effective against *R. solani* are constitutively expressed as it has been observed for biocontrol features of different *Bacillus* or *Trichoderma* biocontrol strains [81,82,83]. A recent study elaborates the transcriptional response of the biocontrol strain *P. fluorescens* In5 to *R. solani*. The authors found no significant changes in global gene expression but noticed that genes possibly involved in metabolite detoxification are highly upregulated [15]. Based on the results obtained in this study, we evaluated the *P. brassicacearum* 3Re2-7 transcriptional profiles of both treatments in a combined manner (see Table 5 to Table 8). 780 genes were determined as highly transcribed, of these 68 as exceptionally highly transcribed, based on the log-2 transformed TPM distribution (Figure 5 and Figure S2).

Among the exceptionally highly transcribed genes is ELZ14_28620, encoding the pyrroloquinoline quinone precursor peptide PqqA. PQQ, a bacterial redox active cofactor for numerous alcohol and aldose dehydrogenases, is derived from the two amino acids glutamate and tyrosine encoded in the precursor peptide PqqA [84]. Moreover, as before-mentioned, it is an important cofactor in inorganic phosphate solubilization and is associated with antifungal activity and induction of systemic resistance [85]. The *pqqA* gene is part of the *pqqABCDEF* operon mentioned before. The other genes of this operon were also transcribed but at moderate rates (log2-transformed TPMs of 3.98 to 6.8).

The four genes ELZ14_06515-30 are all among the top 68 highly expressed genes. They include the three genes encoding structural enzymes of the arginine deiminase (ADI) pathway: carbamate kinase, ornithine carbamoyltransferase and arginine deiminase. In prokaryotes, the biosynthesis of the amino acid arginine, plays a significant role in nitrogen metabolism [86]. Also, arginine and its precursors are involved in the biosynthesis of several metabolites such as polyamines and some antibiotics [87].

Interestingly, four genes of the *phlABCEDFGH* cluster (*phlD*, *C*, *A* and *G*) needed for the production of the antibiotic 2,4-DAPG are among the 68 exceptionally highly transcribed genes.

Further, among the top 68 are ELZ14_09515-30 and ELZ14_09515, which encode subunits of cbb3-type cytochrome-c oxidase. Cytochrome cbb3 oxidases are found almost exclusively in *Proteobacteria* and represent a distinctive class of proton-pumping respiratory heme-copper oxidases (HCO) which are expressed only under microaerobic conditions and thought to be required for colonisation of anoxic tissues [88].

Operon detection applying the ReadXplorer software [89] yielded 1,161 operons (co-transcribed sets of genes resulting in a single polycistronic messenger RNA). Genes were regarded to constitute an operon if a minimum of five spanning reads in sense direction connected the genes. The largest operon harboured 20 genes (ELZ_02445-02540) coding for *inter alia* enzymes that are associated with fatty acid and lipid biosynthesis and translocation of lipoproteins from the inner membrane to the outer membrane. The operon also includes an aromatic amino acid lyase gene that may be involved in the biosynthesis of a wide variety of secondary metabolites such as flavonoids, furanocoumarin phytoalexins and cell wall components [51].

The *hcnABC* cluster, which is one of the named biocontrol determinants (Table 5), also was reported as operon. Further, it was shown to be stongly transcribed (log2-transformed TPM > 9).

Of the *phlABCDEFGH* cluster (Table 5), the *phlABC* operon was reported. The *phlABC* operon encodes a three-protein complex to catalyze the first step of 2,4-DAPG biosynthesis [90]. *PhlABC* and *phlD* show high transcription (log2-transformed TPM > 10).

Interestingly, the *acoABC* genes (Table 6) that encode the subunits of acetoin dehydrogenase were reported to be transcribed in an operon including a 2,3-butanediol dehydrogenase (ELZ14_17085) and an ATP-NAD kinase gene (ELZ14_17105). NAD and NADH are cofactors of 2,3-butanediol dehydrogenase [91]. 2,3-Butanediol dehydrogenase was shown to be able to catalyze the irreversible reduction of diacetyl to (S)-acetoin in *Corynebacterium glutamicum*. Acetoin (3-hydroxy-2-butanone) is a ketone, which is known to be produced by several PGPRs and has been shown to promote plant growth [92]. The transcription rate of this operon is moderate (log2-transformed TPM of 4.5).

The riboflavin biosynthetic pathway (RBP) genes are clustered in operons in most bacteria but their genetic organization varies among species [93,94]. In *P. brassicacearum* 3Re2-7, next to a single *ribBA* locus and a single *ribF* locus, a cluster of RBP genes in the order *ribFAHBED* is present (Table 6). Of these, operon detection reported *ribB*, *ribE* and *ribD* to be transcribed in an operon with a transcriptional regulator NrdR (ELZ14_27890). The association of bacterial *rib* genes with this transcriptional regulator has been observed previously [94].

Some biocontrol determinants of *P. brassicacearum* 3Re2-7 could be produced constitutively. This might be advantageous to sustain in competitive environments like soil and endorhiza. The presence of multiple potentially antifungal gene products points to a synergistic mode of action, as it was shown for *Bacillus amyloliquefaciens* FZB42 [8]. Therefore, the construction of multigenetic mutants in combination with metabolomic analyses could aid the understanding of cooperativity regarding biocontrol traits.

## 3. Materials and Methods

### 3.1. PacBio Library Preparation, Sequencing and Genome Assembly

Genomic DNA was purified from *P. brassicacearum* 3Re2-7 (kindly provided by Dr. Rita Grosch, IGZ Großbeeren, Germany) grown in LB medium to the late-logarithmic growth phase by applying the Gentra Puregene Yeast/Bacteria Kit as outlined in the manual provided by the supplier (Qiagen, Hilden, Germany). The extracted DNA further was teated with the Zymo Genomic DNA Clean & Concentrator${}^{TM}$-10 kit. SMRTbell${}^{TM}$ template library was prepared according to the instructions from PacificBiosciences (Menlo Park, CA, USA) following the ’Procedure & Checklist – Greater Than 10 kb Template Preparation’. Briefly, for preparation of 15 kb libraries, 8 μg genomic DNA was sheared using g-tubes${}^{TM}$ (Covaris, Woburn, MA, USA) according to the manufacturer’s instructions. DNA was end-repaired and ligated overnight to hairpin adapters applying components from the DNA/Polymerase Binding Kit P6 from Pacific Biosciences (Menlo Park, CA, USA). Reactions were carried out according to the instructions of the manufacturer. BluePippin${}^{TM}$ Size-Selection to greater than 4 kb was performed according to the manufacturer’s instructions (Sage Science, Beverly, MA, USA). Conditions for annealing of sequencing primers and binding of polymerase to purified SMRTbell${}^{TM}$ template were assessed with the Calculator in RS Remote (PacificBiosciences, Menlo Park, CA, USA). SMRT sequencing was carried out on the PacBio RSII (PacificBiosciences, Menlo Park, CA, USA) taking one 240-min movie for one SMRT cell using the P6 Chemistry. Sequencing resulted in 90,452 post-filtered reads with a mean read length of 9920 bp. SMRT Cell data was assembled using the “RS_HGAP_Assembly.3” protocol included in SMRT Portal version 2.3.0 using default parameters. The assembly revealed a circular chromosome. Validity of the assembly was checked using the “RS_Bridgemapper.1” protocol. The chromosome was circularized, particularly artificial redundancies at the ends of the contigs were removed and adjusted to *dnaA* as the first gene. Error-correction was performed by a mapping of 1.5 Million paired-end MiSeq Illumina reads of 2 × 300 bp onto the finished genome using BWA [95] with subsequent variant and consensus calling using VarScan [96]. A consensus concordance of QV60 could be confirmed for the genome. The genome sequence was deposited in NCBI GenBank under Accession Number CP034725.

### 3.2. Genome Annotation and Mining

Annotation was carried out using the NCBI Prokaryotic Genome Annotation Pipeline (PGAP) [97]. PHASTER [28] was used to identify prophage sequences, ResFinder-3.1 [29] to detect antimicrobial resistance genes. Genomic Islands (GIs) were detected and visualized in IslandViewer 4 [30]. A screening for bacteriocins was performed applying BAGEL4 [98] (http://bagel4.molgenrug.nl/). For the identification of secondary metabolites clusters antiSMASH 5 beta was used [99]. Predicted NRPS products were searched in the NORINE database [100].

### 3.3. Comparative Analysis

All publicly available genomes of *Pseudomonas brassicacearum* strains reported before June 2019 (Table 1) were obtained in GenBank format from the NCBI genome database (https://www.ncbi.nlm.nih.gov/genome). *P. kilonensis* strains were also included, since this species has previously been suggested as the ’junior synonym’ to *P. brassicacearum* [35]. Metadata of the strains was derived from the associated publications if available (Table 1). Further, genomes of type strains of the genus *Pseudomonas* as named in the list of prokaryotic names with standing in nomenclature (http://www.bacterio.net) were obtained from the NCBI genome database in GenBank format. Namely, the type strain of the genus, *P. aeruginosa* DSM 50071${}^{T}$ [101] and several type strains belonging to the *Pseudomonas fluorescens* group (*P. fluorescens* DSM 50090${}^{T}$ [101], *P. veronii* DSM 11331${}^{T}$ [102], *P. azotoformans* NBRC 12693${}^{T}$ [101], *P. trivialis* DSM 14937${}^{T}$ [103], *P. orientalis* DSM 17489${}^{T}$ [104] and *P. synxantha* NBRC 3913${}^{T}$ [101]) were selected for phylogenetic analysis. Accession numbers of all used strains are listed in Supplementary Table S1. The annotated genomes in GenBank format were used for core genome calculation, construction of a phylogenetic tree, singleton gene analysis and calculation of Average Amino-acid Identity as implemented in EDGAR 2.0 [42]. For calculation of a phylogenetic tree, genomes of known biocontrol strains were integrated. The tree including 33 genomes was built on basis of 2331 core genes per genome. Core genes were aligned using MUSCLE [105] and concatenated to one alignment. FastTree software (http://www.microbesonline.org/fasttree/) was used to generate an approximately-maximum-likelihood phylogenetic tree. All the nodes have bootstrap values of 100. The phylogenetic tree constructed in EDGAR 2.0 was exported in newick format and visualized using Evolview v2 [106]. The DDH estimates were calculated using the Genome-To-Genome Distance Calculator [107] chosing the recommended formula 2. BRIG [31] was used for visualizing the comparison of a reference sequence to a set of query sequences.

### 3.4. Transcriptome Analysis

Cultures were grown on Waksman agar containing 5 g/L of Peptone (Roth, Karlsruhe, Germany), 10 g/L glucose (VWR, Darmstadt, Germany), 3 g/L meat extract (Roth), 5 g/L NaCl (Oxoid, Basingstoke, UK), 20 g/L Agar-Agar Kobe I (Roth) and distilled water (to 1 L), pH 6,8. The strain 3Re2-7 was grown without and with confrontation with *Rhizoctonia solani* AG1-IB isolate 7/3/14 [108]. Each treatment included four replicates which were used to determine the *Pseudomonas brassicacearum* 3Re2-7 transcriptome. After incubation in the dark at room temperature for 36 h, samples of bacterial material were stored in RNAprotect bacterial reagent (Qiagen, Valencia, CA, USA). RNA extraction was done using the RNeasy Mini Kit (Qiagen, Valencia, CA, USA). RNA quality was assessed using the Agilent 2100 bioanalyzer (Agilent Technologies, Palo Alto, CA, USA). The cDNA library generation was performed according to Pátek et al., 2013 [109]. For sequencing, advisements for RNA-Seq profiling of bacterial transcriptomes as stated in Haas et al., 2012 [110] were considered. The cDNA library was sequenced using TruSeq stranded mRNA on the Illumina MiSeq system generating 2 × 75 bp paired-end reads. Obtained RNA-Seq reads were adapter and quality trimmed by means of BBDuk (Bushnell, http://jgi.doe.gov/data-and-tools/bbtools/). Fastx_reverse_complement from FASTX-Toolkit (http://hannonlab.cshl.edu/fastx_toolkit/) was used to maintain the sense direction of transcripts. Then the reads were mapped onto the *P. brassicacearum* 3Re2-7 genome using BBMap [111]. Transcriptome analysis was done with ReadXplorer 2 [89], for calculation of read counts and Transcript Per Million (TPM) values, for operon detection and feature coverage analysis the ‘Single Perfect Match’ and ‘Single Best Match’ options were used. The transcription profiles from both treatments were combined for analysis, since differential gene expression analysis with DESeq2 [112] using the cutoffs p-adjusted value < 0.05 and log2 fold change > 2 yielded no differentially expressed genes. Genes were presumed non-transcribed if the log2-transformed TPM value was below zero, all genes with positive values were considered as verified. Based on the distribution of log2-transformed TPM-values (Figure 5 and Figure S2), genes were assumed highly transcribed, if their log2-transformed TPM value was greater than seven (corresponding non-transformed TPM > 128). Finally, operon detection was performed with a minimum of five spanning reads.

## 4. Conclusions

The strain *P. brassicacearum* 3Re2-7 was selected for genome sequencing due to its biocontrol and plant growth promoting properties [24,25,26,27,32]. Additionally, the strain is commercially applied as plant growth promoting biostimulant. We established its genome sequence as a basis for genome- mining, comparative genomics and transcriptional profiling. We detected putative genetic biocontrol determinants, as well as potentially new biocontrol related genes and gene clusters by genome-mining. Based on the now known genome sequence, the predicted functions of the identified genes and transcripts can be investigated by, for example, mutagenesis experiments. Through comparative analysis, we found unique gene clusters which represent interesting targets for mutagenesis experiments in order to elucidate their function. Our data demonstrated that in *P. brassicacearum* 3Re2-7 biocontrol genes including genes involved in secondary metabolite production are transcribed. These findings underpin its potential as microbial biocontrol agent. Therefore, this study contributes to the basic research towards a safe use of indigenous as well as inoculated *Pseudomonas brassicacearum* biocontrol agents for a sustainable disease management in agriculture.

## Figures and Tables

**Table 1:** General genome features of publicly available genomes of *Pseudomonas brassicacearum* and *P. kilonensis* strains. https://www.ncbi.nlm.nih.gov/genome/genomes/3640, last accessed 13.06.2019.

Organism/Name	Strain	Size [Mb]	GC [%]	Scaf-Folds	Genes	Niche/Source	References	AAI ${}^{a}$
*P. brassicacearum*	3Re2-7	6.7	60.8	1	6267	Potato endorhiza	[32], this study	99.9
*P. brassicacearum* subsp. *brassicacearum*	NFM421 ${}^{b}$	6.8	60.8	1	6209	*Arabidopsis thaliana* rhizoplane	[16,18]	100
*P. brassicacearum*	DF41	6.6	60.5	1	5884	Canola root	[19]	96
*P. brassicacearum*	LBUM300	6.9	60.8	1	6308	Strawberry rhizosphere	[22]	99.9
*P. brassicacearum*	L13-6-12	6.7	60.9	1	6046	Potato rhizosphere	[21]	99.9
*P. brassicacearum*	BS3663	6.7	60.8	1	6129	No metadata available	Unpublished	99.9
*P. brassicacearum*	51MFCVI2.1	6.5	61.0	49	5980	Rhizosphere	Unpublished	99.9
*P. brassicacearum*	PP1_210F	6.7	60.8	5	6159	Peels tuber	[20,38]	99.9
*P. brassicacearum*	PA1G7	6.7	60.8	8	6180	Potato field soil	[20,38]	99.9
*P. brassicacearum*	LZ-4	6.2	60.1	111	5642	Yellow River	Unpublished	94.9
*P. brassicacearum*	TM1A3	6.6	60.9	29	6037	Tomato rhizosphere	[39]	99.8
*P. brassicacearum*	Wood3	6.3	62.3	433	5752	Agricultural	Unpublished	94.7
*P. brassicacearum*	36B7	7.1	60.7	28	6411	Agricultural	Unpublished	95.8
*P. brassicacearum*	36D4	7.0	60.5	128	6290	Agricultural	Unpublished	95.8
*P. brassicacearum*	Delaware	6.8	60.8	226	6263	Agricultural	Unpublished	99.8
*P. brassicacearum*	93F8	6.8	61.0	36	6291	Agricultural	Unpublished	99.9
*P. brassicacearum*	37D10	6.3	58.8	55	5946	Agricultural	Unpublished	88.6
*P. brassicacearum*	Wood1	6.9	60.8	187	6288	Agricultural	Unpublished	99.8
*P. brassicacearum*	48H11	6.0	58.8	36	5510	Agricultural	Unpublished	88.7
*P. brassicacearum*	38D7	6.5	59.5	485	6087	Agricultural	Unpublished	89.2
*P. brassicacearum*	38D4	7.1	58.7	199	6793	Agricultural	Unpublished	89.5
*P. kilonensis*	1855-344	6.8	60.7	73	6146	Soil	[40]	98.2
*P. kilonensis*	P12	6.4	60.8	44	5791	Tobacco	[39]	98.2
*P. kilonensis*	DSM 13647${}^{T}$	6.4	60.9	44	5758	No metadata available	Unpublished	98.1
*P. kilonensis*	ACN7	6.5	61	200	5897	Compost soil	[41]	98.2
*P. kilonensis*	ACN4	6.5	60.8	91	5956	Compost soil	[41]	98.2
*P. kilonensis*	ZKA7	6.8	60.6	1	6158	No metadata available	Unpublished	97.9
*P. kilonensis*	BS3780	6.4	60.8	2	5775	No metadata available	Unpublished	98.1

${}^{a}$ Average Amino-acid Identity (AAI) with the representative strain of the *P. brassicacearum* species, NFM421; ${}^{b}$ Representative strain.

**Table 2:** Annotation of *P. brassicacearum* 3Re2-7 singleton cluster_1.

Locus tag	Predicted Function
ELZ14_04045	hypothetical protein
ELZ14_04050	DNA-binding protein
ELZ14_04055	hypothetical protein
ELZ14_04060	hypothetical protein
ELZ14_04065	hypothetical protein
ELZ14_04070	hypothetical protein
ELZ14_04075	nucleotidyltransferase family protein
ELZ14_04080 *	hypothetical protein
ELZ14_04085 *	MafI family immunity protein
ELZ14_04090	hypothetical protein
ELZ14_04095	hypothetical protein
ELZ14_04100	hypothetical protein
ELZ14_04105	DUF4935 domain-containing protein
ELZ14_04110	hypothetical protein
ELZ14_04115 *	hypothetical protein
ELZ14_04120	hypothetical protein
ELZ14_04125 *	hypothetical protein
ELZ14_04130 *	hypothetical protein
ELZ14_04135 *	integrase
ELZ14_04140	hypothetical protein
ELZ14_04145	hypothetical protein
ELZ14_04150	hypothetical protein
ELZ14_04155	hypothetical protein
ELZ14_04160	metallohydrolase

* Non-singleton genes.

**Table 3:** Annotation of *P. brassicacearum* 3Re2-7 singleton cluster_2.

Locus Tag	Predicted Function
ELZ14_15030	hypothetical protein
ELZ14_15035 *	hypothetical protein
ELZ14_15040	helicase IV
ELZ14_15045 *	HNH endonuclease
ELZ14_15050	hypothetical protein
ELZ14_15055	endonuclease
ELZ14_15060	hypothetical protein
ELZ14_15065 *	hypothetical protein
ELZ14_15070	hypothetical protein
ELZ14_15075	hypothetical protein
ELZ14_15080	hypothetical protein
ELZ14_15085	CHAT domain-containing protein
ELZ14_15090	hypothetical protein
ELZ14_15095 *	DNA/RNA non-specific endonuclease
ELZ14_15100 *	hypothetical protein
ELZ14_15105 *	relaxase
ELZ14_15110	DUF3742 family protein
ELZ14_15115 *	conjugal transfer protein TraG
ELZ14_15120 *	integrating conjugative element protein
ELZ14_15125 *	TIGR03756 family integrating conjugative element protein
ELZ14_15130 *	DNA repair protein RadC
ELZ14_15135	hypothetical protein
(...) 32 *	
ELZ14_15295	HAD family hydrolase
ELZ14_15300	DNA-processing protein DprA
ELZ14_15305 *	hypothetical protein
ELZ14_15310	hypothetical protein
ELZ14_15315	DUF4935 domain-containing protein
ELZ14_15320	hypothetical protein
ELZ14_15325	DUF3800 domain-containing protein
ELZ14_15330	toll/interleukin-1 receptor domain-containing protein
ELZ14_15335	RNA-directed DNA polymerase
ELZ14_15340	hypothetical protein
ELZ14_15345 *${}^{,a}$	hypothetical protein
ELZ14_15350 *${}^{,a}$	transposase
ELZ14_15355	hypothetical protein
ELZ14_15360 *	hypothetical protein
ELZ14_15365	hypothetical protein
ELZ14_15370 *	hypothetical protein
ELZ14_15375	ATP-binding protein
ELZ14_15380 *	hypothetical protein
ELZ14_15385 *${}^{,a}$	leucine ABC transporter subunit substrate-binding protein LivK
ELZ14_15390	hypothetical protein

* Non-singleton genes; ${}^{a}$ Pseudo gene.

**Table 4:** Annotation of *P. brassicacearum* 3Re2-7 singleton cluster_5.

Locus Tag	Predicted Function
ELZ14_26355	hypothetical protein
ELZ14_26360	hypothetical protein
ELZ14_26365	DNA helicase
ELZ14_26370	GNAT family N-acetyltransferase

**Table 5:** Genetic biocontrol determinants of *P. brassicacearum* 3Re2-7—Secondary Metabolism and Antibiotics.

Biocontrol Trait	Locus Tag (Gene Name)	Predicted EC Number and Protein Function	log2(TPM ${}^{a}$)
**Secondary metabolism & antibiotics**			
	ELZ14_11930 (*phlE*)	n.a.; MFS transporter	8.18
	ELZ14_11935 (*phlD*)	EC 2.3.1.-; type III polyketide synthase	11.05
	ELZ14_11940 (*phlB*)	n.a.; 2,4-diacetylphloroglucinol biosynthesis protein	10.12
DAPG ${}^{b}$ synthesis	ELZ14_11945 (*phlC*)	n.a.; thiolase family protein	10.79
	ELZ14_11950 (*phlA*)	EC 2.3.3.10; hydroxymethylglutaryl-CoA synthase	10.86
	ELZ14_11955 (*phlF*)	n.a.; TetR/AcrR family transcriptional regulator	5.23
	ELZ14_11960 (*phlG*)	n.a.; 2,4-diacetylphloroglucinol hydrolase	10.97
	ELZ14_11965 (*phlH*)	n.a.; TetR/AcrR family transcriptional regulator	7.07
	ELZ14_17910 (*hcnC*)	EC 1.4.99.5; cyanide-forming glycine dehydrogenase subunit HcnC	9.33
HCN ${}^{c}$ synthesis	ELZ14_17915 (*hcnB*)	EC 1.4.99.5; cyanide-forming glycine dehydrogenase subunit HcnB	8.95
	ELZ14_17920 (*hcnA*)	EC 1.4.99.5; cyanide-forming glycine dehydrogenase subunit HcnA	9.89

${}^{a}$ Transcript Per Million; ${}^{b}$ 2,4-diacetylphloroglucinol; ${}^{c}$ Hydrogen cyanide.

**Table 6:** Genetic biocontrol determinants of *P. brassicacearum* 3Re2-7—Induced systemic resistance & Plant growth promotion.

Biocontrol Trait	Locus Tag (Gene Name)	Predicted EC Number and Protein Function	log2 (TPM ${}^{a}$)
	ELZ14_17085 (-)	EC 1.1.1.4; 2,3-butanediol dehydrogenase	4.47
	ELZ14_17090 (*acoC*)	EC 2.3.1.12; acetoin dehydrogenase dihydrolipoyllysine-residue acetyltransferase subunit	4.51
	ELZ14_17095 (*acoB*)	EC 1.1.1.-; alpha-ketoacid dehydrogenase subunit beta	4.55
Volatiles	ELZ14_17100 (*acoA*)	EC 1.1.1.-; thiamine pyrophosphate-dependent dehydrogenase E1 component subunit alpha	4.58
	ELZ14_26505 (*ilvC*)	EC 1.1.1.86; Ketol-acid reductoisomerase	8.25
	ELZ14_26510 (*ilvH*)	EC 2.2.1.6; Acetolactate synthase isozyme 3 small subunit	6.78
	ELZ14_26515 (*ilvI*)	EC 2.2.1.6; Acetolactate synthase isozyme 3 large subunit	6.13
	ELZ14_12055 (*ilvX*)	EC 2.2.1.6; Putative acetolactate synthase large subunit IlvX	1.81
	ELZ14_11285 (*acdS*)	EC 3.5.99.7; 1-aminocyclopropane-1-carboxylate deaminase	4.66
	ELZ14_14325 (*ribBA*)	n.a.; 3,4-dihydroxy-2-butanone-4-phosphate synthase/GTP cyclohydrolase II	1.98
	ELZ14_23780 (*ribH*)	EC 2.5.1.78; 6,7-dimethyl-8-ribityllumazine synthase	4.92
	ELZ14_26870 (*ribF*)	EC 2.7.1.26; bifunctional riboflavin kinase/FAD synthetase	5.40
	ELZ14_27845 (*ribA*)	EC 3.5.4.25; GTP cyclohydrolase II	5.63
	ELZ14_27870 (*ribH2*)	EC 2.5.1.78; 6,7-dimethyl-8-ribityllumazine synthase	8.05
Plant growth promotion	ELZ14_27875 (*ribB*)	n.a.; 3,4-dihydroxy-2-butanone-4-phosphate synthase	4.82
	ELZ14_27880 (*ribE*)	EC 2.5.1.9; riboflavin synthase	4.69
	ELZ14_27885 (*ribD*)	n.a.; riboflavin biosynthesis protein RibD	3.80
	ELZ14_28390 (*trpC*)	EC 4.1.1.48; indole-3-glycerol phosphate synthase TrpC	6.23
	ELZ14_28395 (*trpD*)	EC 2.4.2.18; anthranilate phosphoribosyltransferase	5.98
	ELZ14_28400 (*trpG*)	EC 4.1.3.27; aminodeoxychorismate/anthranilate synthase component II	5.02
	ELZ14_28405 (*estA*)	EC 3.1.1.1; autotransporter domain-containing esterase	3.90
	ELZ14_28410 (*trpE*)	EC 4.1.3.27; anthranilate synthase component I	4.43
	ELZ14_15680 (*phyC*)	EC 3.1.3.8; phytase	0.18
	ELZ14_27855 (*pgpA*)	EC 3.1.3.27; phosphatidylglycerophosphatase A	6.49
	ELZ14_28415 (*gph*)	EC 3.1.3.18; phosphoglycolate phosphatase	4.60
	ELZ14_28615 (*pqqF*)	EC:3.4.24.-; pyrroloquinoline quinone biosynthesis protein PqqF	3.98
	ELZ14_28620 (*pqqA*)	n.a.; pyrroloquinoline quinone precursor peptide PqqA	12.77
	ELZ14_28625 (*pqqB*)	n.a.; pyrroloquinoline quinone biosynthesis protein PqqB	6.86
	ELZ14_28630 (*pqqC*)	EC 1.3.3.11; pyrroloquinoline-quinone synthase PqqC	5.99
Phosphate solubilization	ELZ14_28635 (*pqqD*)	n.a.; pyrroloquinoline quinone biosynthesis peptide chaperone PqqD	6.32
	ELZ14_28640 (*pqqE*)	n.a.; pyrroloquinoline quinone biosynthesis protein PqqE	5.96
	ELZ14_30735 (-)	EC 3.1.3.16; serine/threonine-protein phosphatase	5.10
	ELZ14_08470 (*mupP*)	EC 3.1.3.105; N-acetylmuramic acid 6-phosphate phosphatase MupP	7.05
	ELZ14_08475 (*ubiG*)	EC 2.1.1.222; 3-demethylubiquinol 3-O-methyltransferase UbiG	7.36
	ELZ14_04440 (-)	EC 3.1.3.1; alkaline phosphatase	6.63
	ELZ14_04880 (-)	EC 3.1.3.1; alkaline phosphatase family protein	2.57
	ELZ14_09815 (-)	EC 3.1.3.16; phosphoprotein phosphatase	3.15

${}^{a}$ Transcript Per Million.

**Table 7:** Genetic biocontrol determinants of *P. brassicacearum* 3Re2-7 - Pathogen inhibition.

Biocontrol Trait	Locus Tag (Gene Name)	Predicted EC Number and Protein Function	log2 (TPM ${}^{a}$)
	ELZ14_01095 (-)	n.a.; TonB-dependent siderophore receptor	0.32
	ELZ14_04245 (*fur*)	n.a.; ferric iron uptake transcriptional regulator	8.94
	ELZ14_10605 (-)	n.a.; TonB-dependent siderophore receptor	1.14
	ELZ14_12290 (*hcsK*)	n.a.; siderophore-iron reductase FhuF	4.09
	ELZ14_13330 (*pchB*)	n.a.; isochorismate lyase	4.10
	ELZ14_14280 (-)	n.a.; TonB-dependent siderophore receptor	2.35
	ELZ14_16995 (-)	n.a.; TonB-dependent siderophore receptor	1.85
Iron acquisition	ELZ14_18660 (-)	n.a.; TonB-dependent siderophore receptor	0.84
	ELZ14_21210 (*pvdA*)	EC 1.14.13.196; ornithine monooxygenase	1.30
	ELZ14_21260 (-)	n.a.; nonribosomal peptide synthetase	1.42
	ELZ14_21280 (-)	n.a.; TonB-dependent siderophore receptor	2.82
	ELZ14_21285 (-)	n.a.; nonribosomal peptide synthetase	3.41
	ELZ14_24950 (-)	n.a.; siderophore-interacting protein	4.42
	ELZ14_26060 (-)	n.a.; TonB-dependent siderophore receptor	0.26
	ELZ14_28775 (-)	n.a.; TonB-dependent siderophore receptor	0.46
	ELZ14_14600 (-)	n.a.; matrixin family metalloprotease	12.59
	ELZ14_14610 (*prsD*)	n.a.; type I secretion system permease/ATPase PrsD	5.81
Exoprotease activity	ELZ14_14615 (-)	n.a.; HlyD family type I secretion periplasmic adaptor subunit	6.32
	ELZ14_14620 (-)	n.a.; peptidase	4.73
	ELZ14_20130 (-)	n.a.; Hcp family type VI secretion system effector	1.77
Chitinase activity	ELZ14_25035 (*nagA*)	EC 3.5.1.25; N-acetylglucosamine-6-phosphate deacetylase	2.72

${}^{a}$ Transcript Per Million.

**Table 8:** Genetic biocontrol determinants of *P. brassicacearum* 3Re2-7—Others.

Biocontrol Trait	Locus Tag (Gene Name)	Predicted EC Number and Protein Function	log2 (TPM ${}^{a}$)
	ELZ14_02400 (*gltB*)	EC 1.4.1.13; glutamate synthase large subunit	5.12
	ELZ14_02405 (*gltD1*)	EC 1.4.1.13; glutamate synthase small subunit	5.59
	ELZ14_02380 (*aroK*)	EC 2.7.1.71; shikimate kinase AroK	5.44
Metabolism	ELZ14_02385 (*aroB*)	EC 4.2.3.4; 3-dehydroquinate synthase	5.04
	ELZ14_03505 (*aroQ*)	EC 4.2.1.10; type II 3-dehydroquinate dehydratase	5.88
	ELZ14_08510 (*aroA*)	EC 2.5.1.19; 3-phosphoshikimate 1-carboxyvinyltransferase	6.40
	ELZ14_11900 (-)	n.a.; polyketide cyclase	2.67
	ELZ14_25290 (*algD*)	n.a.; nucleotide sugar dehydrogenase	9.65
	ELZ14_25300 (*alg44*)	n.a.; alginate biosynthesis protein Alg44	7.31
	ELZ14_25320 (*algX*)	n.a.; alginate O-acetyltransferase	7.49
	ELZ14_25325 (*algL*)	EC 4.2.2.3; mannuronate-specific alginate lyase	7.89
Exopolysaccharides	ELZ14_25335 (*algJ*)	n.a.; alginate O-acetyltransferase	7.22
	ELZ14_25340 (*algF*)	n.a.; alginate O-acetyltransferase	8.93
	ELZ14_25350 (*algA*)	n.a.; alginate biosynthesis protein AlgA	10.73
	ELZ14_27635 (*sacB*)	EC 2.4.1.10; glycoside hydrolase 68 family protein (levansucrase)	6.33
	ELZ14_00175 (*katE*)	EC 1.11.1.6; catalase HPII	8.02
	ELZ14_03625 (*copA*)	EC 3.6.3.54; copper-exporting P-type ATPase A	3.70
	ELZ14_03915 (*katG*)	EC 1.11.1.21; catalase/peroxidase HPI	6.31
	ELZ14_09480 (-)	EC 3.6.3.54; cadmium-translocating P-type ATPase	5.50
Detoxification	ELZ14_24395 (*copA*)	n.a.; copper resistance system multicopper oxidase	2.25
	ELZ14_24400 (*copB*)	n.a.; copper resistance protein B	1.40
	ELZ14_24405 (*copC*)	n.a.; Copper resistance protein C	3.03
	ELZ14_24410 (*copD*)	n.a.; Copper resistance D family protein	2.07
	ELZ14_24510 (*dps2*)	EC 1.16.-.-; DNA starvation/stationary phase protection protein	9.59
	ELZ14_27055 (*katB*)	EC 1.11.1.6; catalase	2.63
Lipopolysaccharides	ELZ14_31170 (-)	n.a.; lipoteichoic acid (LTA) synthase family protein	1.48

${}^{a}$ Transcript Per Million.

## Supplementary Materials

The following are available online at https://www.mdpi.com/2073-4425/10/8/601/s1. Figure S1: By antiSMASH predicted core structure of the putative NRPS encoded by sM_r9. Figure S2: Distribution of log2-transformed Transcript Per Million (TPM) values of all genes. Table S1: Accession numbers of all strains used in bioinformatic analyses.
