# Supplementary figures and images for: Genetic Potential of the Biocontrol Agent Pseudomonas brassicacearum (Formerly P. trivialis) 3Re2-7 Unraveled by Genome Sequencing and Mining, Comparative Genomics and Transcriptomics
Source: Genes (Basel). 2019 Aug 9;10(8):601. doi: 10.3390/genes10080601 (PMC6722718; doi:10.3390/genes10080601)

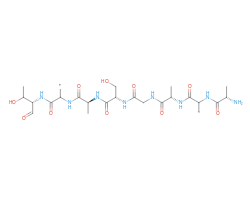

Supplement: Supplementary file 1 [file genes-10-00601-s001.zip › Figure_S1_predictedCoreStructure_sM_r9.png]

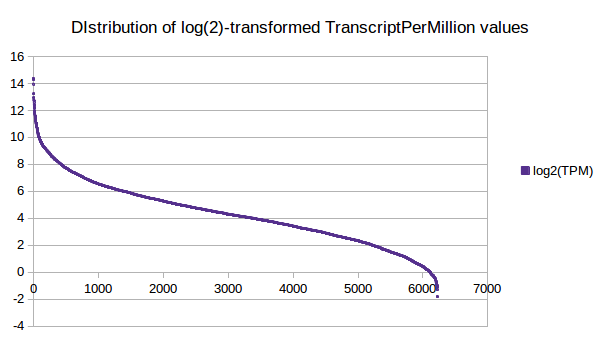

Supplement: Supplementary file 1 [file genes-10-00601-s001.zip › Figure_S2_TPM_distribution.png]
